# Association between magnesium intake and the risk of anemia among adults in the United States

**DOI:** 10.3389/fnut.2023.1046749

**Published:** 2023-02-23

**Authors:** Jungao Huang, Jing Xu, Ping Ye, Xiaoqin Xin

**Affiliations:** ^1^Ganzhou Maternal and Child Health Hospital, Ganzhou, Jiangxi, China; ^2^Department of Clinical Laboratory, Ganzhou People’s Hospital, Ganzhou, Jiangxi, China

**Keywords:** magnesium intake, anemia, association, adult, risk

## Abstract

**Background:**

Magnesium deficiency is related to an increased risk of anemia, but epidemiological evidence supporting this association remains scarce. The purpose of the present survey was to evaluate the relationship between dietary magnesium intake and the risk of anemia.

**Methods:**

In total, 13,423 participants aged 20–80 years were enrolled using data from the National Health and Nutrition Examination Survey 2011–2016. Magnesium consumption was evaluated using 24 h dietary recalls. Multivariable generalized linear models were developed to demonstrate the association between dietary magnesium intake and the prevalence of anemia.

**Results:**

An inverse association between dietary magnesium intake and the risk of anemia was detected based on a full adjustment model. We evaluated magnesium intake as a categorical variable (five quartiles). Compared with the lowest value, the highest multivariate adjusted odds ratio (95% confidence interval) for anemia was 0.64 (0.46–0.89). Stratified analyses revealed a reverse relationship between magnesium intake and anemia in women. However, no significant association was observed in men (*p*_for trend_ = 0.376). A similar reverse association was found among the older group (aged ≥60 years).

**Conclusion:**

Magnesium deficiency is closely related to a higher rate of anemia occurrence, especially among women and older Americans. Further larger-scale prospective studies are required to confirm these conclusions.

## 1. Introduction

Anemia threatens public health worldwide. The prevalence of anemia nearly doubled (4.0 to 7.1%) from 2003–2004 to 2011–2012 (1). Anemia is associated with an increased sequence of adverse effects, including cardiovascular disease, low quality of life, morbidity, and mortality (2–4). Anemia reflects the decreased oxygen-carrying capacity of the blood, which may lead to fatigue, cardiovascular complications, and impaired body function (5–7). Moreover, anemia has been shown to increase hospitalization rates, especially in older adults (8).

Magnesium plays a crucial role in the functioning and sustainment of the body (9). The imbalance of magnesium homeostasis can lead to modification of the cell membrane and increased oxidative stress (10). Magnesium deficiency often leads to inflammation through the activation of the nuclear factor kappa B (NF-kB) pathway in immune cells and in the pathogenesis of many chronic disorders, including congestive heart failure, type 2 diabetes and hypertension (11, 12). In recent decades, a few studies have indicated that magnesium is involved in the regulation of cell replication, differentiation, and apoptosis (13–15). Magnesium is important for the hematopoietic system (16). In the United States, dietary magnesium intake is often below the recommended dietary intake, and 28% of women develop anemia during pregnancy due to magnesium deficiency (17). A cross-sectional retrospective study by Zeynep et al. identified a positive relationship between magnesium deficiency and anemia among individuals with chronic kidney disease (18). Moreover, Cinar et al. reported that magnesium supplementation increases hemoglobin levels in athletes (19).

Although these studies have reported that magnesium deficiency has a potentially modifiable association with anemia, they have mostly focused on specific populations. Data on the relationship between dietary magnesium intake and anemia in the general population are limited. Therefore, we explored the association of magnesium intake with anemia in adults, as well as possible effects of age and sex, using data from the National Health and Nutrition Examination Survey (NHANES) database between 2011 and 2016.

## 2. Materials and methods

### 2.1. Study population

Data were collected from three continuous survey cycles (between 2011 and 2016) of the NHANES, which was a nationally representative survey conducted by the Centers for Disease Control and Prevention (CDC) and the National Center for Health Statistics (NCHS) to estimate health and nutrition in the US population. The NHANES contains demographic, nutritional, and medical examination information of civilian noninstitutionalized people in the US. Written informed consent was provided by all participants, and the survey protocol was supported by the Institutional Review Board of the NCHS (20).

**Figure 1:**
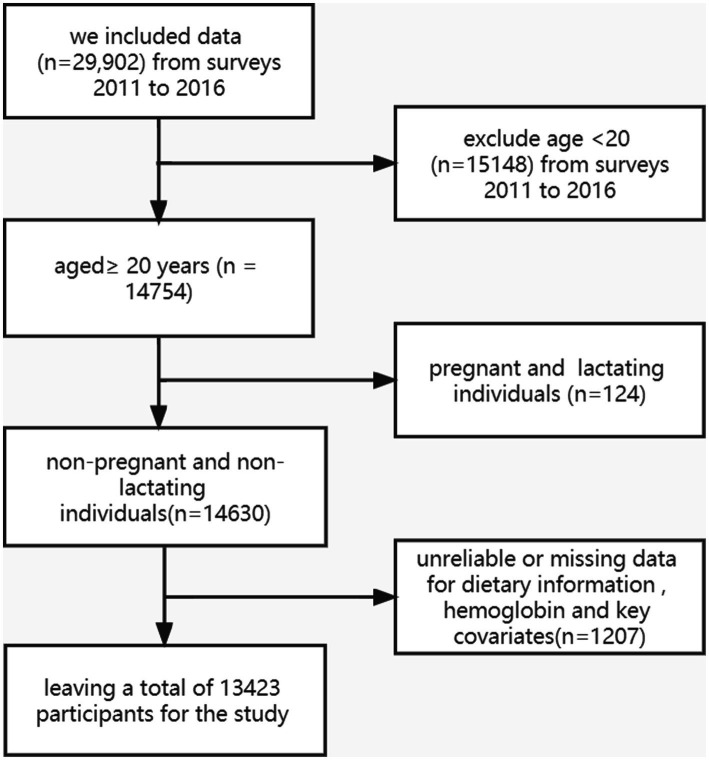
Flowchart of recruitment in our study.

Data of 14,754 individuals aged 20–80 years from 2011 to 2016 were extracted. Pregnant or lactating females (*n* = 124) and participants with unreliable or missing magnesium intake data, hemoglobin data, and important confounders were further excluded (*n* = 1,207). Ultimately, 13,423 participants were included in the analysis (Figure 1).

### 2.2. Nutrient intake assessments

Nutritional information including total dietary energy, vitamin D, calcium, magnesium, protein, and fiber intakes was collected *via* the first 24 h dietary recall interview, which was performed at the Mobile Examination Center (MEC). Data collection by 24-h recall interview is the most common method used to determine dietary intake in large-scale surveys and has been used in the NHANES for many years, based on expert consensus (21). Details of the dietary interview have been described in the Dietary Interviewers Procedure Manuals (22). The dietary interview information included food species, consumption frequency, duration, and quantity. Information on dietary intake is detailed in the NHANES.^1^

### 2.3. Anemia assessment

Anemia was described as a hemoglobin concentration < 120 g/L for women and < 130 g/L for men (23).

### 2.4. Other covariates

Covariates included age, sex, race/ethnicity, educational experience, smoking status, physical activity level, and body mass index (BMI). Dietary information included total energy, protein, fiber, magnesium, calcium, and Vitamin D intakes. Race was classified into the following categories: Mexican American, non-Hispanic Black, non-Hispanic White, Other Hispanic, and Other Race. Educational background was categorized into “less than a high school diploma,” “graduated from high school,” or “education beyond high school” category. Poverty income ratio (PIR) was calculated as the federal poverty level divided by the family income and defined as a value <1 or ≥ 1. BMI was estimated as weight in kilograms divided by height in meters squared and categorized into <25.0, 25.0 to <30.0, and ≥ 30.0 kg/m^2^. The participants were classified as current smokers, former smokers, and never smokers according to their smoking status. Physical activity was categorized into three strata: light activity, moderate activity, and vigorous activity. Dietary data, including total dietary energy, vitamin D, calcium, magnesium, protein, and fiber intakes, were acquired from a 24-h dietary recall interview.

### 2.5. Statistical analysis

Statistical analysis was conducted using the statistical software package R^2^ and Empower Stats (X&Y Solutions, Inc., Boston, MA, United States).^3^ Categorical variables and continuous variables were evaluated using chi-square tests and t-tests, respectively. Generalized linear models were developed to explore the connection between magnesium intake and anemia. First, we used univariable logistic regression to identify factors linked to anemia. Second, multivariate logistic regression models were applied. In the crude model, we made no adjustments. Model 1 was adjusted for sex, age and race. Model 2 was adjusted for all confounders listed in Table 1, including age; sex; race; PIR value; educational level; BMI; smoking status; physical activity level; and dietary energy, protein, fiber, vitamin D, calcium, and magnesium intakes. Third, a series of sensitivity analyses were conducted to identify the robustness of the results. Magnesium intake was divided into five quartiles to test the p for trends, and the lowest quartile was considered the reference. Finally, to explore the robustness of our results, analyses were stratified by sex and age as shown in Tables 2, 3. The results were considered statistically significant at *p* < 0.05.

**Table 1:** Characteristics of participants by anemia status, NHANES 2011–2016.

Variables	Total (*n* = 13,423)	Non-anemia (*n* = 11,947)	Anemia (*n* = 1,476)	*P* value
**Gender (%)**				<0.001
Female	6,745 (50.2)	5,813 (48.7)	932 (63.1)	
Male	6,678 (49.8)	6,134 (51.3)	544 (36.9)	
**Age (%)**				<0.001
20–44	5,601 (41.7)	5,122 (42.9)	479 (32.5)	
45–59	3,339 (24.9)	3,040 (25.4)	299 (20.3)	
≥60	4,483 (33.4)	3,785 (31.7)	698 (47.3)	
**Race (%)**				<0.001
Mexican American	1,842 (13.7)	1,649 (13.8)	193 (13.1)	
Non-Hispanic Black	2,938 (21.9)	2,305 (19.3)	633 (42.9)	
Non-Hispanic White	5,380 (40.1)	5,021 (42)	359 (24.3)	
Other Hispanic	1,431 (10.7)	1,303 (10.9)	128 (8.7)	
Other race	1,832 (13.6)	1,669 (14)	163 (11)	
**PIR (%)**				<0.001
<1	2,750 (22.2)	2,391 (21.7)	359 (26.8)	
≥1	9,621 (77.8)	8,639 (78.3)	982 (73.2)	
Education level (%)				<0.001
College education or above	2,908 (21.7)	2,509 (21)	399 (27)	
Graduated from high school	2,967 (22.1)	2,644 (22.1)	323 (21.9)	
<High school	7,545 (56.2)	6,791 (56.9)	754 (51.1)	
**BMI (kg/m^2^) (%)**				0.047
<25	3,809 (28.6)	3,396 (28.6)	413 (28.7)	
25–30	4,316 (32.4)	3,887 (32.8)	429 (29.8)	
≥30	5,181 (38.9)	4,584 (38.6)	597 (41.5)	
Energy(kcal), Median (IQR)	1942.0 (1438.0, 2588.0)	1966.0 (1465.0, 2626.0)	1734.0 (1252.0, 2280.0)	<0.001
Protein(kcal), Median (IQR)	74.0 (52.5, 100.9)	75.2 (53.5, 102.7)	64.7 (45.9, 88.4)	<0.001
Fiber(kcal), Median (IQR)	14.8 (9.6, 22.0)	15.0 (9.8, 22.3)	13.0 (8.4, 19.8)	<0.001
Calcium(mg), Median (IQR)	802.0 (517.0, 1175.0)	816.0 (529.0, 1190.0)	690.5 (443.8, 1053.0)	<0.001
Vitamin D(mg), Median (IQR)	3.0 (1.1, 5.9)	3.1 (1.2, 6.0)	2.8 (1.1, 5.5)	0.002
Magnesium(mg), Median (IQR)	270.0 (194.0, 369.0)	274.0 (198.0, 374.0)	239.0 (166.0, 327.0)	<0.001
**Smoker status (%)**				<0.001
Never smoker	7,572 (56.5)	6,659 (55.8)	913 (61.9)	
Former smoker	3,199 (23.9)	2,827 (23.7)	372 (25.2)	
Current smoker	2,639 (19.7)	2,449 (20.5)	190 (12.9)	
**Work activity (%)**				<0.001
Light work activity	8,042 (59.9)	7,037 (58.9)	1,005 (68.1)	
Moderate work activity	2,781 (20.7)	2,484 (20.8)	297 (20.1)	
Vigorous work activity	2,598 (19.4)	2,424 (20.3)	174 (11.8)	

**Table 2:** Odds ratios (95% confidence intervals) of anemia across quartiles of dietary magnesium intake stratify by gender, NHANES 2011–2016.

Magnesium intake (mg/d)	N Event%	Crude model		Model 1		Model 2	
		OR (95%CI)	*P* value	OR (95% CI)	*P* value	OR (95%CI)	*P* value
Female							
Log2 Mg	932 (13.8)	0.73 (0.67 ~ 0.81)	<0.001	0.79 (0.71 ~ 0.87)	<0.001	0.71 (0.58 ~ 0.86)	0.001
Q1 (<179)	307 (18)	1(Ref)	<0.002	1(Ref)		1(Ref)	
Q2 (179–239)	194 (12.6)	0.66 (0.54 ~ 0.8)	<0.003	0.71 (0.58 ~ 0.87)	0.001	0.73 (0.58 ~ 0.92)	0.007
Q3 (239–304)	191 (13.1)	0.69 (0.56 ~ 0.83)	<0.004	0.76 (0.62 ~ 0.93)	0.008	0.78 (0.6 ~ 1.01)	0.058
Q4 (304–395)	158 (13)	0.68 (0.55 ~ 0.84)	<0.005	0.78 (0.63 ~ 0.97)	0.026	0.76 (0.55 ~ 1.05)	0.096
Q5 (>395)	82 (9.8)	0.5 (0.38 ~ 0.64)	<0.006	0.57 (0.44 ~ 0.74)	<0.001	0.56 (0.36 ~ 0.87)	0.01
P for trend			<0.007		<0.001		0.046
Male							
Log2 Mg	544 (8.1)	0.65 (0.58 ~ 0.72)	<0.001	0.82 (0.72 ~ 0.93)	0.002	0.9 (0.7 ~ 1.15)	0.401
Q1 (<179)	127 (12.9)	1(Ref)		1(Ref)		1(Ref)	
Q2 (179–239)	107 (9.7)	0.72 (0.55 ~ 0.95)	0.019	0.85 (0.63 ~ 1.14)	0.266	0.89 (0.64 ~ 1.24)	0.479
Q3 (239–304)	106 (8.5)	0.62 (0.47 ~ 0.82)	0.001	0.82 (0.61 ~ 1.09)	0.174	0.89 (0.62 ~ 1.27)	0.509
Q4 (304–395)	100 (6.8)	0.49 (0.37 ~ 0.65)	<0.001	0.7 (0.52 ~ 0.94)	0.019	0.84 (0.56 ~ 1.25)	0.385
Q5 (>395)	104 (5.6)	0.4 (0.3 ~ 0.52)	<0.001	0.67 (0.5 ~ 0.89)	0.007	0.78 (0.46 ~ 1.32)	0.354
P for trend			<0.001		0.003		0.376

Crude model adjusted for none. Model 1 adjusted for age and race. Model 2 adjusted for all covariates listed in Table 1.

**Table 3:** Odds ratios (95% confidence intervals) of anemia across quartiles of dietary magnesium intake stratified by age NHANES 2011–2016.

Magnesium intake (mg/d)	N (Event %)	Crude model		Model 1		Model 2	
		OR (95% CI)	*P* value	OR (95% CI)	*P* value	OR (95 %CI)	*P* value
Age (20–44)							
Q1 (<179)	134 (12.6)	1(Ref)		1(Ref)		1(Ref)	
Q2 (179–239)	89 (8.6)	0.65 (0.49 ~ 0.87)	0.003	0.73 (0.54 ~ 0.98)	0.039	0.82 (0.59 ~ 1.14)	0.244
Q3 (239–304)	97 (8.5)	0.64 (0.49 ~ 0.85)	0.002	0.78 (0.58 ~ 1.05)	0.098	0.91 (0.64 ~ 1.3)	0.607
Q4 (304–395)	91 (8.3)	0.63 (0.47 ~ 0.83)	0.001	0.95 (0.7 ~ 1.29)	0.751	1.25 (0.83 ~ 1.89)	0.291
Q5 (>395)	68 (5.4)	0.39 (0.29 ~ 0.53)	<0.001	0.81 (0.58 ~ 1.12)	0.203	1.05 (0.61 ~ 1.8)	0.867
P for trend			<0.001		0.526		0.392
Age (45–60)							
Q1 (<179)	77 (12.9)	1(Ref)		1(Ref)		1(Ref)	
Q2 (179–239)	60 (9.5)	0.71 (0.5 ~ 1.01)	0.059	0.79 (0.55 ~ 1.15)	0.219	0.84 (0.56 ~ 1.25)	0.387
Q3 (239–304)	63 (9.8)	0.74 (0.52 ~ 1.05)	0.088	0.93 (0.64 ~ 1.34)	0.698	0.97 (0.63 ~ 1.51)	0.908
Q4 (304–395)	54 (7.4)	0.54 (0.37 ~ 0.78)	0.001	0.75 (0.52 ~ 1.11)	0.148	0.72 (0.43 ~ 1.21)	0.217
Q5 (>395)	45 (6.2)	0.44 (0.3 ~ 0.65)	<0.001	0.67 (0.44 ~ 1)	0.051	0.6 (0.3 ~ 1.19)	0.141
P for trend			<0.001		0.065		0.206
Age (>60)							
Q1 (<179)	223 (21.8)	1(Ref)		1(Ref)		1(Ref)	
Q2 (179–239)	152 (15.5)	0.66 (0.52 ~ 0.83)	<0.001	0.73 (0.57 ~ 0.92)	0.007	0.78 (0.59 ~ 1.02)	0.07
Q3 (239–304)	137 (14.8)	0.63 (0.49 ~ 0.79)	<0.001	0.67 (0.53 ~ 0.86)	0.001	0.74 (0.54 ~ 1.01)	0.055
Q4 (304–395)	113 (13.3)	0.55 (0.43 ~ 0.7)	<0.001	0.6 (0.46 ~ 0.77)	<0.001	0.63 (0.43 ~ 0.92)	0.017
Q5 (>395)	73 (10.4)	0.41 (0.31 ~ 0.55)	<0.001	0.42 (0.31 ~ 0.56)	<0.001	0.44 (0.26 ~ 0.75)	0.003
P for trend			<0.001		<0.001		0.005

Crude model adjusted for none. Model 1 adjusted for sex and race. Model 2 adjusted for all covariates listed in Table 1.

## 3. Results

Sociodemographic characteristics and possible confounding factors grouped by anemia status are shown in Table 1. In total, 13,423 individuals were included in this sample, and 1,476 participants (11%) were defined as having anemia. Compared with participants without anemia, those with anemia were more likely to be female, older, non-Hispanic black, individuals with a lower daily dietary intake (magnesium, calcium, vitamin D, energy, fiber, and protein), those with a lower family income, and individuals with obesity.

The multivariate logistic regression analysis results are displayed in Table 4. We determined an inversely proportional association between dietary magnesium intake (log2 transformation) and the risk of anemia in the crude model (OR, 0.66; 95% CI, 0.61–0.7). In Model 1, we adjusted for age, sex, and race. The OR (95% CI) was 0.78 (0.72–0.84). After adjusting for all possible confounders, the result was compatible with that of Model 1 (OR, 0.78; 95% CI, 0.68–0.91). We divided magnesium intake into five quartiles. The p for trends was robust irrespective of the three different model analyses (crude model: *p* < 0.001, Model 1: *p* < 0.001, Model 2: *p* = 0.035).

**Table 4:** Odds ratios (95% confidence intervals) of anemia across quartiles of dietary magnesium intake.

Magnesium intake (mg/d)	Event (%)	Crude model		Model 1		Model 2	
		OR (95% CI)	*P* value	OR (95% CI)	*P* value	OR (95% CI)	*P* value
Magnesium (log2 transform)	1,476 (11)	0.66 (0.61~0.7)	<0.001	0.78 (0.72~0.84)	<0.001	0.78 (0.68~0.91)	0.002
Q1 (<179)	434 (29.40)	1(Ref)		1(Ref)		1(Ref)	
Q2 (179–239)	301 (20.39)	0.65 (0.56~0.76)	<0.001	0.72 (0.61~0.84)	<0.001	0.76 (0.63~0.92)	0.008
Q3 (239–304)	297 (20.12)	0.63 (0.54~0.74)	<0.001	0.76 (0.64~0.9)	0.002	0.83 (0.67~1.02)	0.097
Q4 (304–395)	258 (17.48)	0.55 (0.46~0.64)	<0.001	0.71 (0.6~0.84)	<0.001	0.78 (0.61~1)	0.066
Q5 (>395)	186 (12.60)	0.38 (0.32~0.45)	<0.001	0.56 (0.47~0.68)	<0.001	0.64 (0.46~0.89)	0.011
P for trend			<0.001		<0.001		0.035

Crude model adjusted for none. Model 1 adjusted for age, gender and race. Model 2 adjusted for all covariates listed in Table 1.

Stratified analyses for dietary magnesium intake by sex were conducted (Table 2). Among female participants, magnesium consumption was inversely associated with anemia (*p* for trend = 0.046). Nevertheless, this correlation did not show a significant difference in males (*p* for trend = 0.376).

We further conducted age-stratified analyses to evaluate the association of magnesium intake with anemia (Table 3). After adjusting for all confounders, an inverse relationship between dietary magnesium intake and the risk of anemia was statistically remarkable among the older group (age ≥ 60 years [*p* for trend = 0.005]). However, there was no statistically significance in the other two groups (age < 60 years). To ensure the robustness of our findings, a subgroup analysis was conducted to evaluate the potential interaction between magnesium intake and anemia (Table 5). After adjustment for age, sex, race, PIR value, educational level, BMI, smoking status, physical activity level, and dietary intake of energy, protein, fiber, vitamin D, and calcium, our smoking status had an interactive effect on the relationship between daily magnesium intake and anemia (*p* for interaction = 0.012). Non-interactive effect was calculated in other subgroup (*p* for interaction <0.05).

**Table 5:** Subgroups analysis of association between diet magnesium intake and anemia.

Subgroup	n.total	n.event_%	Crude OR 95CI	Crude P value	Adj OR 95CI	Adj P value	P for interaction
**Gender**							0.427
Female	6,745	932 (13.8)	0.73 (0.67 ~ 0.81)	<0.001	0.71 (0.58 ~ 0.86)	0.001	
Male	6,678	544 (8.1)	0.65 (0.58 ~ 0.72)	<0.001	0.9 (0.7 ~ 1.15)	0.401	
**Age**							0.57
20–44	4,482	333 (7.4)	0.64 (0.56 ~ 0.74)	<0.001	0.88 (0.65 ~ 1.19)	0.405	
45–59	4,458	445 (10)	0.69 (0.6 ~ 0.79)	<0.001	0.75 (0.58 ~ 0.98)	0.037	
≥60	4,483	698 (15.6)	0.68 (0.61 ~ 0.76)	<0.001	0.77 (0.61 ~ 0.98)	0.036	
**Race**							0.168
Mexican American	1842	193 (10.5)	0.81 (0.66 ~ 1)	0.052	1.04 (0.65 ~ 1.67)	0.869	
Non-Hispanic Black	2,938	633 (21.5)	0.66 (0.58 ~ 0.74)	<0.001	0.71 (0.57 ~ 0.9)	0.004	
Non-Hispanic White	5,380	359 (6.7)	0.75 (0.65 ~ 0.87)	<0.001	1.03 (0.76 ~ 1.39)	0.848	
Other Hispanic	1,431	128 (8.9)	0.63 (0.49 ~ 0.81)	<0.001	0.58 (0.36 ~ 0.96)	0.032	
Other race	1832	163 (8.9)	0.72 (0.57 ~ 0.9)	0.005	0.74 (0.46 ~ 1.19)	0.218	
**Poverty-income ratio**							0.233
PIR < 1	2,750	359 (13.1)	0.74 (0.65 ~ 0.85)	<0.001	0.92 (0.7 ~ 1.22)	0.577	
PIR ≥ 1	9,621	982 (10.2)	0.62 (0.57 ~ 0.68)	<0.001	0.73 (0.61 ~ 0.88)	0.001	
**Education level**							0.197
College education or above	2,908	399 (13.7)	0.69 (0.61 ~ 0.8)	<0.001	0.98 (0.73 ~ 1.32)	0.886	
Graduated from high school	2,967	323 (10.9)	0.74 (0.63 ~ 0.86)	<0.001	0.67 (0.48 ~ 0.92)	0.013	
< High school	7,545	754 (10)	0.62 (0.56 ~ 0.69)	<0.001	0.77 (0.62 ~ 0.94)	0.013	
**BMI**							0.051
<25	3,809	413 (10.8)	0.67 (0.59 ~ 0.77)	<0.001	0.77 (0.58 ~ 1.01)	0.057	
25–30	4,316	429 (9.9)	0.58 (0.51 ~ 0.67)	<0.001	0.67 (0.5 ~ 0.88)	0.004	
≥30	5,181	597 (11.5)	0.71 (0.64 ~ 0.8)	<0.001	0.89 (0.7 ~ 1.13)	0.333	
**Smoking status**							0.012
Never smoker	7,572	913 (12.1)	0.6 (0.54 ~ 0.66)	<0.001	0.73 (0.6 ~ 0.89)	0.002	
Former smoker	3,199	372 (11.6)	0.61 (0.53 ~ 0.7)	<0.001	0.77 (0.57 ~ 1.03)	0.08	
Current smoker	2,639	190 (7.2)	0.87 (0.73 ~ 1.05)	0.153	1.07 (0.75 ~ 1.53)	0.716	
**Physical activity, n (%)**							0.237
Light work activity	8,042	1,005 (12.5)	0.67 (0.62 ~ 0.73)	<0.001	0.79 (0.66 ~ 0.95)	0.01	
Moderate work activity	2,781	297 (10.7)	0.61 (0.51 ~ 0.72)	<0.001	0.79 (0.56 ~ 1.11)	0.174	
Vigorous work activity	2,598	174 (6.7)	0.74 (0.6 ~ 0.91)	0.004	0.91 (0.59 ~ 1.4)	0.654	

OR, odds ratio; CI, confidence interval; BM, Body Mass Index; Diet magnesium intake were log2 transformed. Adjusted for age, sex, race, PIR value, educational level, BMI, smoking status, physical activity level, and dietary energy, protein, fiber, vitamin D and calcium.

## 4. Discussion

In the present cross-sectional survey, an inverse association between dietary magnesium intake and anemia was found among US adults, utilizing data from three continued NHANES cycles. In the sex-stratified analysis, an inverse association was found in females, whereas no significant difference was observed in males. Furthermore, we noticed a similar relationship between dietary magnesium intake and the risk of anemia among older participants (age ≥ 60 years). To the best of our knowledge, this is the first and largest sociodemographic investigation to reveal the relationship between magnesium intake and the prevalence of anemia in a general population.

The recommended daily allowance for magnesium intake in US adults is 420 mg for males and 320 mg for females. However, our data showed that daily magnesium intake value was 239 mg in the anemia group, which is significantly lower than recommended daily allowance. Inadequate magnesium intake has been a growing concern in recent years. Magnesium deficiency has been partially attributed to unhealthy dietary pattern such as the consumption of so-called “Western diet” (24, 25). Clinical magnesium deficiency or magnesium deficiency patients can be found in internal medicine. Magnesium deficiency has been associated with a number of diseases, including atherosclerosis (26), diabetes (27), hypertension (28), myocardial infarction (29), and calculi (30). Nuts, fresh vegetables, and integral grains are the major sources of magnesium. Moreover, with the exception of milk, the concentration of magnesium in dairy products is very low (24). Therefore, the consumption of foods rich in magnesium may decrease the risk of certain diseases.

A limited number of studies have quantified the relationship between magnesium intake and anemia. A similar study found an inverse correlation between magnesium intake and anemia depending on ferritin levels among 8,511 Chinese adults. However, this association was not significant with serum ferritin levels <15 ng/mL (25). A cross-sectional domestic review of 2,849 Chinese adults aged 20 years or older reported that sufficient magnesium and iron intakes were positively correlated with hemoglobin levels and negatively linked to the prevalence of anemia (31). Another similar study involving 2,401 individuals aged 60 years or older in China showed that adopting a modern dietary pattern (magnesium consumption) is an appropriate strategy for preventing anemia in older Chinese people (32). In addition, an investigation indicated that low levels of magnesium and serum ferritin were linked to a higher risk of anemia among 180 pregnant women from Khartoum, Sudan (33). Our study extended these findings in a much larger cohort (*n* = 13,423) and different subgroups.

Although the precise potential mechanism of this association between magnesium and anemia remains unclarified, several possible mechanisms may explain our results. Magnesium is considered an important coenzyme for glutathione peroxidase, which is involved in the synthesis of hemoglobin (34, 35). Furthermore, animal experiments have shown that magnesium deficiency can cause microcytic anemia, damage the membranes of red blood cells, and reduce the osmotic fragility of erythrocytes in rats (36–38). Magnesium deficiency reduces erythrocyte energy metabolism and hemoglobin synthesis, leading to anemia (25). Moreover, chronic magnesium deficiency may promote the release of inflammatory compounds (39). Moreover, a study reported that anemia is more prevalent in individuals with hemodialysis who suffer from decreasing erythropoietin (EPO) concentrations; nevertheless, increased serum magnesium level appears to reduce the risk of anemia by enhancing EPO response (40). In addition, higher concentrations of magnesium may promote the driving of HIF-1α (hypoxia-inducible factor) expression, which is mediated by reactive oxygen species (ROS) *via* the NF-κB signaling pathway, in which HIFs are considered an important factor in the process of hemoglobin production (41, 42). A supposed mechanism is that magnesium deficiency may alter macrophage and iron homeostasis through the NF-κB pathway, which may indirectly impair the membranes and accelerate the aging of and damage to RBCs (43).

This study had several advantages. (1) To our knowledge, this was the first study to investigate the relationship between dietary magnesium intake and anemia using a nationally representative sample of US adults. (2) This was the largest investigation exploring this association, which may ensure statistical efficiency. (3) We controlled and adjusted for more potential confounders. (4) A sensitivity analysis stratified by sex and age was performed to explore potential special populations. However, our study had certain limitations. First, in consideration of the characteristics of cross-sectional studies, the temporal sequence of this relationship could not be assessed. Second, biochemical parameters, serum magnesium, and the type of anemia information were not available in the database. In addition, multiple 24-h dietary recalls could not signify long-term magnesium status. Finally, our study was vulnerable to unmeasured confounders. Therefore, more large sample prospective studies are needed to further probe the mechanisms of the relationship between magnesium and anemia.

In conclusion, magnesium deficiency is positively associated with a higher rate of anemia occurrence, especially among females and older populations. Healthy and adequate dietary magnesium intake should be promoted.

## Data availability statement

The raw data supporting the conclusions of this article will be made available by the authors, without undue reservation.

## Ethics statement

The studies involving human participants were reviewed and approved by the Institutional Review Board of the NCHS. The patients/participants provided their written informed consent to participate in this study.

## Author contributions

JH drafted the manuscript. JX and PY collected the clinical data. XX conceived the study. All authors read and approved the final manuscript.

## Conflict of interest

The authors declare that the research was conducted in the absence of any commercial or financial relationships that could be construed as a potential conflict of interest.

## Publisher’s note

All claims expressed in this article are solely those of the authors and do not necessarily represent those of their affiliated organizations, or those of the publisher, the editors and the reviewers. Any product that may be evaluated in this article, or claim that may be made by its manufacturer, is not guaranteed or endorsed by the publisher.

## Footnotes

^1^ https://www.cdc.gov/nchs/nhanes

^2^ http://www.R-project.org

^3^ http://www.empowerstats.com
